# Nitric Oxide in Cerebral Vasospasm: Theories, Measurement, and Treatment

**DOI:** 10.1155/2013/972417

**Published:** 2013-06-25

**Authors:** Michael Siuta, Scott L. Zuckerman, J. Mocco

**Affiliations:** ^1^Vanderbilt University, School of Medicine, 121 Medical Center Drive, Nashville, TN 37232, USA; ^2^Department of Neurological Surgery, Vanderbilt University, School of Medicine, Medical Center Drive, Nashville, TN 37232, USA

## Abstract

In recent decades, a large body of research has focused on the role of nitric oxide (NO) in the development of cerebral vasospasm (CV) following subarachnoid hemorrhage (SAH). Literature searches were therefore conducted regarding the role of NO in cerebral vasospasm, specifically focusing on NO donors, reactive nitrogen species, and peroxynitrite in manifestation of vasospasm. Based off the assessment of available evidence, two competing theories are reviewed regarding the role of NO in vasospasm. One school of thought describes a deficiency in NO due to scavenging by hemoglobin in the cisternal space, leading to an NO signaling deficit and vasospastic collapse. A second hypothesis focuses on the dysfunction of nitric oxide synthase, an enzyme that synthesizes NO, and subsequent generation of reactive nitrogen species. Both theories have strong experimental evidence behind them and hold promise for translation into clinical practice. Furthermore, NO donors show definitive promise for preventing vasospasm at the angiographic and clinical level. However, NO augmentation may also cause systemic hypotension and worsen vasospasm due to oxidative distress. Recent evidence indicates that targeting NOS dysfunction, for example, through erythropoietin or statin administration, also shows promise at preventing vasospasm and neurotoxicity. Ultimately, the role of NO in neurovascular disease is complex. Neither of these theories is mutually exclusive, and both should be considered for future research directions and treatment strategies.

## 1. Introduction

Subarachnoid hemorrhage (SAH) is a form of stroke that affects 28,000 individuals in North America each year [1]. A frequent cause of SAH is the rupture of an intracranial aneurysm, leading to extravasation of blood into the subarachnoid space. While aneurysmal SAH accounts for only 7% of all cerebrovascular accidents (CVAs), those that suffer SAH have an average age of 51 years, significantly younger than those with a thromboembolic or hemorrhagic stroke [1]. Due to the young age of these patients, they have great potential to return to their premorbid state and level of productivity, with successful intervention. However, even with endovascular or surgical repair of the offending aneurysm, those that survive the initial insult can still accumulate additional neurologic defects in the days and weeks post-hemorrhage. Enormous efforts to understand and prevent additional mortality following SAH led to the discovery of the phenomenon known as cerebral vasospasm (CV) [1].

CV refers to the constriction of smooth muscle in blood vessels feeding the brain, leading to reductions in blood flow to downstream brain parenchyma. CV occurs angiographically on days 4–7 post-SAH, with narrowing of the arteries of the circle of Willis in 40% to 70% of patients [1]. Clinical manifestations of vasospasm generally follow a characteristic progression, beginning with mental status changes, proceeding to motor and speech impairments, and culminating in permanent neurological damage or death [1]. Vasospasm is hypothesized to underlie the neurologic deficits that occur after SAH, given that the deficits seen follow a similar time course as the development of angiographic vasospasm [1]. However, it is important to note that other mechanisms are likely to contribute to long-term outcome following SAH, including cortical spreading depression, generation of microthrombemboli, pathologic vascular changes, and generation of neurotoxic intermediates [2–9]. Additionally, vasospasm of vessels supplying noneloquent areas of cortex may not affect clinical manifestations of vasospasm but may affect long-term outcomes.

The study of CV remains an important focus in preventing complications following SAH. One promising mechanism to prevent clinical vasospasm involves enhancement of nitric oxide (NO) signaling in the cerebral vasculature [10]. NO is a gaseous signaling molecule critical for maintaining blood vessel patency and promoting local hemodynamics [4]. NO is synthesized by three different enzymes in the body, and its synthesis is rapidly upregulated following aneurysmal rupture [11]. Additionally, the regulation of NO is important for a range of physiological events outside of blood flow regulation. An improved understanding of NO can, therefore, provide insight into additional mechanisms underlying neurologic damage after SAH and direct future interventions.

Thus, the objective of our review is to (1) assess the physiological role of NO in control of cerebral blood flow, (2) discuss the evidence supporting NO in the pathophysiology of SAH, at the level of vasospastic collapse and oxidative damage, and (3) report the experimental and clinical findings regarding interventions for SAH, ranging from enhancement of NO signaling directly to indirect stimulation of NO synthase. We hope to assess the strength of evidence and enhance our scientific understanding of the role of NO in CV following SAH.

## 2. Role of Nitric Oxide in Cerebral Blood Flow

### 2.1. NO and CBF

The central nervous system has the greatest blood flow of any human organ. As the brain does not have energy storage capacity of its own, it needs to maintain continuous nutrient and oxygen delivery in order to support its metabolic demand. Therefore, when blood flow to the central nervous system is disrupted, as occurs in the acute period following SAH and several days later during CV, permanent neurologic damage can ensue. Elaborate mechanisms have evolved to maintain constant blood flow to the CNS, mechanisms that are sensitive to changes in mean arterial blood pressure, cerebral perfusion pressure, pH, and pCO_2_ [12]. Emerging evidence suggests that NO is one such molecule that has evolved to support cerebral blood flow (CBF) during periods of rest and changing metabolic demand, such as alteration in synaptic activity, activation of vasodilator perivascular nerves, and hypercapnia. The activity of NO, and therefore the effects of NO on CBF, can be controlled by targeting NO signaling cascades and NO synthesis [12].

### 2.2. NO Signaling

NO was discovered as an endothelium-derived relaxing factor in 1986. NO signals through activation of soluble guanylyl cuyclase (sGC), a heme-containing enzyme located on the inside surface of the cell membrane. sGC then acts to stimulate formation of cyclic guanine monophosphate (cGMP) from GTP. cGMP acts as a second messenger, signaling through multiple kinases to regulate the function of numerous signaling cascades and ion channels downstream. As a result of sGC/cGMP signaling, NO signaling leads to dephosphorylation of myosin light chains and hyperpolarization of smooth muscle cells, causing relaxation of smooth muscle fibers and dilating cerebral arterioles. NO signaling is terminated through the actions of phophodiesterases (PDEs), which act to break down cGMP intracellularly [4]. 

### 2.3. Synthesis of NO

NO is synthesized by the enzyme nitric oxide synthase (NOS). Its synthesis requires one arginine molecule, two oxygen molecules, and 1.5 NADP+ molecules for electron transfer. The molecule L-citrulline is produced in equimolar amounts with NO, a finding critical when considering how to measure NO. All three of the NOS isoforms require the cofactors tetrahydrobiopterin, FAD, FMN, calmodulin, and heme to function [4, 13]. The heme-containing component of NOS is critical for understanding how hemoglobin released by vascular injury can bind to available NO, precipitating the development of vasospasm. Furthermore, the tetrahydrobiopterin cofactor plays an important role in NOS dysfunction, and subsequent development of harmful reactive oxygen and nitrogen species after SAH [14].

NOS is encoded by three isoforms in mammals: endothelial NOS (eNOS), neuronal NOS (nNOS), and inducible NOS (iNOS). eNOS, as the name implies, is localized primarily in the endothelium. nNOS is located in certain neurons, consistent with a role of NO as a retrograde neurotransmitter, and in perivascular nerves, including those that innervate the adventitial layer of arteries. Both eNOS and nNOS produce NO in a relatively constitutive fashion, in a manner that fluctuates in response to intracellular calcium levels [15]. The function of eNOS and nNOS depends on the binding of these proteins to a calcium-sensing protein called calmodulin [15]. The function of iNOS, in contrast, is not constitutively active. iNOS is expressed in nearly all cell types, including endothelial cells, vascular smooth muscle cells, macrophages, and others [15]. The basal expression of iNOS is very low, but it may be the most critical isoform for NO production after SAH, as its transcription is stimulated by hypoxia and inflammatory cascades, and it produces roughly 100–1000 times the NO of its counterparts, in a manner independent of calcium [15]. All three NOS isoforms are influenced by SAH. Both eNOS and iNOS phosphorylation are potently stimulated after SAH [11], and nNOS levels markedly decrease, perhaps due to degeneration of nNOS neurons after SAH [16]. 

### 2.4. NO Metabolism and Reactive Oxygen Species

While NO is a critical molecule for normal regulation of cerebral blood flow, its overproduction may have significance in pathological states. NO is a free radical signaling species with a very short half life in brain (between 2 seconds and 2 minutes), after which it is rapidly oxidized to its inactive metabolites nitrite and nitrate. Recent evidence indicates that, under certain physiological conditions, nitrite and nitrate can be reduced back into NO [17–20].

The role of NO as a free radical is important when considering that, in conditions like SAH, the production of NO is going to be markedly upregulated. Overproduction of NO by iNOS has been implicated in many pathophysiological processes caused by ischemia and inflammation. The potential neurotoxic role of NO is incompletely understood, but is hypothesized to depend on interactions between NO and superoxide anion, resulting in the formation of peroxynitrite. SAH provides many sources of free radicals to potentiate the formation of peroxynitrite, involving the stimulation of enzymes including xanthine oxidase, NADPH oxidase, and the arachidonic acid cascade, while inhibiting antioxidant enzymes including superoxide dismutase and glutathione peroxidase [21]. Additionally, animal models demonstrate that SAH disrupts the flow of electrons in the mitochondrial electron transport chain after SAH, providing a potential source of electrons for superoxide formation [22–24]. 

**Figure 1:**
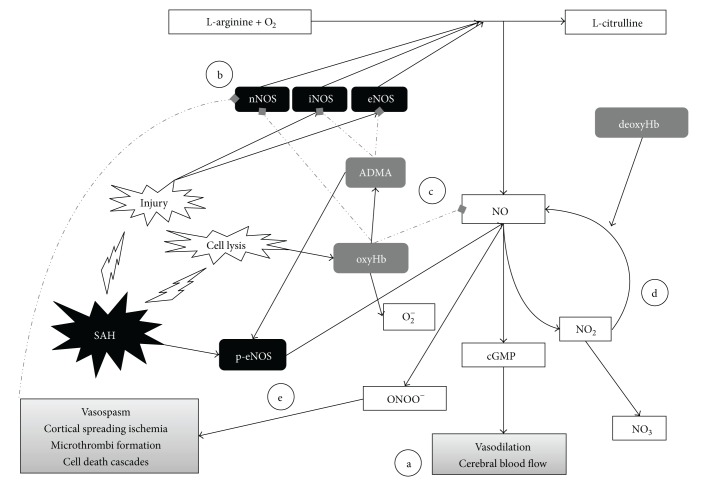
The regulation of nitric oxide after subarachnoid hemorrhage. Schematic demonstrating the production of NO from its precursors and its significance in SAH. Solid arrows depict positive regulatory steps. Dotted, gray arrows depict negative regulatory steps. (a) NO stimulation down the cGMP-mediated signaling pathway is critical for vasodilation and maintenance of cerebral blood flow. All NO donor strategies outlined in the text are intended to stimulate this final common pathway. (b) The three forms of nitric oxide synthases play critical roles in the pathogenesis of SAH and vasospasm. iNOS and eNOS activity are upregulated after SAH. Primate models suggest that nNOS levels decrease after SAH and may precipitate the development of vasospasm, potentially due to Hb-mediated oxidative damage. (c) Classical evidence suggests that NO is scavenged by the Hb that is released into the CSF after SAH-induced vascular injury, as a potential mechanism underlying vasospastic collapse. Hb also may trigger the production of the protein ADMA, an endogenous inhibitor of NOS that is hypothesized to play a major role in vasospasm. (d) NO is metabolized into nitrite (NO_2_) and nitrate (NO_3_), which serve as indirect measurements of NO in clinical and animal models. New evidence suggest that nitrite can be metabolized back into NO by hemoglobin in its deoxygenated form, leading to promising investigations regarding nitrite donors for treatment of SAH. (e) Recent investigations, based to a large extent on animal models, focus on the potential role of eNOS uncoupling in the pathogenesis of vasospasm. eNOS uncoupling occurs after SAH, through a mechanism that may stem from binding by ADMA, micronutrient deficiency, presence of superoxide, or lack of the substrate arginine. The eNOS-catalyzed formation of peroxynitrite from NO and reactive oxygen species may contribute to the development of delayed neurologic damage after SAH.

In the context of SAH, a last, but critical, source of free radicals for reactive oxygen/nitrogen species formation is from hemoglobin itself. Indeed, both oxyhemoglobin and methemoglobin are found in much higher amounts in the CSF of patients afflicted by SAH [25] and have the potential to generate free radical formation, trigger autocatalytic lipid peroxidation cycles, and induce cytotoxic signaling cascades [21, 26–28]. These free radical generating mechanisms are important considerations when constructing therapeutic frameworks for treatment of SAH, and the role of NOS itself in generation of NO free radicals will be considered in later sections. A visual schematic of all signaling pathways involved with NO after SAH is summarized in Figure 1.

### 2.5. Additional Physiological Roles of NO

In addition to the mechanisms mentioned above, there are numerous other effects of NO that may be critical to neurologic outcome after SAH. Evidence from research on cardiovascular disease suggests that NO serves to influence platelet aggregation and adhesion, intimal hyperplasia [29, 30], leukocyte adhesion and migration [30], and smooth muscle proliferation under physiological conditions. Additionally, beyond the role of NO in vasospasm itself, NO may influence other critical mechanisms that contribute to neurologic outcome after SAH, including cortical spreading depression, microthromboemboli formation, and consequences of vascular injury itself [31]. While these processes warrant further investigation and discussion, this review is focused more specifically on the roles of NO in vasodilation and free radical production. The role of NO in vasodilation and free radical production, as it pertains to CV after subarachnoid hemorrhage, will therefore be the focus of subsequent sections.

## 3. Measurement of Nitric Oxide

The half-life of NO in the human body, as mentioned previously, is on the order of seconds to minutes. Due to the difficulties in measurement of NO directly, measurement of nitrite and nitrate, two stable oxidized metabolites of NO, are used instead. In the context of SAH, nitrite and nitrate are most frequently assessed in the CSF of patients who have external ventricular drains placed, although studies have been performed involving direct measurement of NO in brain through placement of microdialysis catheters.

There are conflicting reports in the literature regarding how nitrite and nitrate levels relate to CV. However, there is a general consensus regarding the dynamics of NO after SAH. For the purposes of this review, we will adopt the framework put forth in a recent review by Sehba et al. [32, 33]. Due to difficulties in real world assessment of NO or its metabolites in the period directly after SAH, Sehba and authors focused primarily on animal models for the neurochemical changes in the early period after SAH (between 0 and 60 min). According to the generalized consensus in the animal model literature, there is an early decrease in NO in the period directly following SAH, presumably due to hemoglobin scavenging, consumption by neutrophils, and interaction of NO with free radicals [33]. Phase II, between 1 and 6 hours post-SAH, is characterized by a return of NO function to baseline, presumably due to induction of eNOS and iNOS activity post-SAH [32]. Phase III occurs between 6–72 hours and is characterized by an increase in nitrite/nitrate compared to control conditions [32]. This phase III is based on findings from additional animal models and clinical studies. In terms of clinical studies of patients with SAH, one finding indeed revealed that, compared to a control population which underwent surgical repair of unruptured aneurysms, patients with ruptured SAH had significantly higher levels of nitrite and nitrate in their CSF [34]. Another study supported a relationship between nitrite/nitrate and SAH, demonstrating that these metabolites correlate with oxygen tension in patients with aneurysmal rupture [35].

CSF relationships between NO and vasospasm are less clear than the relationship between SAH and NO. Several CSF studies of NO metabolites in vasospasm patients yielded conflicting results. Suzuki et al. demonstrated decreased levels of nitrite in SAH patients who experienced vasospasm compared to those that did not [36]. Similar findings were demonstrated by Jung et al. a decade later, replicating not only the decreased nitrite levels in patients with vasospasm but also increased levels of an endogenous nitric oxide inhibitor, ADMA, in CV patients. Additionally, levels of ADMA in the CSF were not only lower in patients with vasospasm but also correlated with angiographic determination of vasospasm [37]. However, Rejdak et al. demonstrated a contrary finding, showing that patients with very good outcomes had lower levels of nitrite in the CSF than those with unfavorable outcomes [25]. Supporting Rejdak et al.'s initial finding, Woszczyk et al. additionally suggest that patients with vasospasm after SAH in fact have higher levels of nitrite and nitrate than those without [38]. Rejdak et al. suggest that the mechanism underlying this effect is the ability of NO overproduction to cause oxidative stress and brain injury [25]. 

Therefore, NO in the CSF is not a clear biomarker to assess risk for development of CV. Measurements directly on brain parenchyma are similarly inconsistent. Animal models measuring NO directly in brain parenchyma have shown reduced levels of the NO metabolites nitrite and nitrate in brain tissue immediately after SAH [33], decreased NO availability 2 days post-SAH [39], decreased NO release [11], and decreased staining of nNOS in the adventitial layer of animals succumbing to vasospasm [16]. Microdialysis findings in humans are inconsistent in probing a directionality between NO metabolite levels and CV after SAH. In one microdialysis study, Sakowitz et al. developed a study design to confirm that nitrate and nitrite levels increased in patients who experienced a ruptured SAH compared to patients who did not experience hemorrhage [40]. In their study, however, they found no difference in brain nitrite or nitrate levels between patients who suffered from delayed neurologic deficits and those who did not [40]. Several studies have since been performed involving microdialysis assessments of CV patients, and some findings suggest that increased nitrite/nitrate levels are associated with worse outcomes, while others indicate that there is no difference [40–44]. The variability in the data is likely to depend on a combination of factors, as most of these studies were relatively low powered, and NO is notoriously difficult to measure.

In addition to measurement of NO metabolites, investigations regarding the effects of NO-associated genes on the sensitivity to vasospasm have recently been initiated. Indeed, in one study involving an SAH cohort, individuals with genetic variation in the promoter region of the eNOS gene show a dose-dependent increase in risk for vasospasm after SAH, with heterozygous individuals for a particular risk alleles having a 3.3-fold increased odds of developing CV and homozygotes a 10.9-fold increased risk [45]. However, despite initial enthusiasm for this finding, other findings demonstrate contrasting results, with alternative genetic polymorphisms at the same allelic site demonstrating increased risk of vasospasm [46]. Indeed, these initial studies note that the power to detect effects may be lacking, and the patient populations they studied are heterogeneous. The significance of these genetic studies at a functional level is also unknown. Therefore, prospective studies, meta-analyses, and analyses of the functional effect of these genetic polymorphisms are warranted to appropriately measure the true impact of genetic variability on susceptibility to vasospasm.

While the relationship between nitrite/nitrate and vasospasm therefore is not entirely clear, the net consensus is that there is at first a reduction in NO in the period immediately following SAH, followed by a return to baseline at 1–6 hours, and an increase in NO at 6–72 hours [32]. Progress has been made in terms of establishing a link between NO and vasospasm, and future studies should pay attention to measurement of species such as the endogenous NOS inhibitor ADMA, given the potential for such compounds to affect nitrite and nitrate measurements. Additionally, future efforts aimed at quantification of mechanisms like cortical spreading depression, and microthromboemboli formation may help to understand the roles of NO signaling in neurologic dysfunction after SAH. 

## 4. Theories of Nitric Oxide in Vasospasm

### 4.1. Hemoglobin as an “NO Scavenger”

One of the biggest prognostic indicators of vasospasm post-SAH is the presence of blood in the cisterns [47]. Additionally, it is well known that breakdown products of red blood cells are vasospastic in nature, and that one likely spasmogen is oxyhemoglobin (oxyHb). These same species are elevated manifold in the CSF after SAH. Intriguingly, in line with the presence of heme in the proteins responsible for NO synthesis and signaling, hemoglobin is capable of tightly binding NO, having 1000x more affinity for NO than oxygen. Initial theories regarding the role of NO in vasospasm, therefore, suggested that Hb in the cisterns acts as a “sink” for NO, depriving it of its ability to control normal vascular tone and thus cause vasoconstriction [4]. This basic concept is supported by studies of patients with sickle cell disease, which demonstrate that hemoglobin, when it is outside of red blood cells, acts to reduce the bioavailability of NO [48]. Indeed, experimental evidence *in vivo *demonstrates that NO is scavenged from canine basilar arteries when Hb is applied [49]. As experimental evidence indicates that NO is protective against angiographic vasospasm, and clinical evidence suggests that nitric oxide donors like nitroglycerin improve neurologic outcomes after SAH [4], NO remains an attractive pathway for development of future therapies. 

Apart from the scavenging effect, Hb may contribute to vasospasm after-SAH through generation of reactive oxygen and nitrogen species [4, 50], stimulation of an NOS inhibitor [4], or the metabolism, and subsequent oxidation of hemoglobin to bilirubin to the BOXes complexes, which serve as vasoconstrictive agents [51]. Additionally, a recent body of work suggests that the deoxygenated form of Hb (deoxyHb) may actually serve to promote NO signaling by acting as a nitrite reductase, producing NO from nitrite as a precursor [17]. This potential ability of deoxyhemoglobin to replenish NO in this fashion has led to much research into the potential role of nitrite infusions to prevent neurologic damage after SAH [17–20]. 

### 4.2. NOS Uncoupling and Oxidative Stress

Additional theories gaining popularity in recent years center around dysfunction in NOS itself as the causative event behind CV. Indeed, all forms of NOS are affected by SAH. Upregulation of iNOS and eNOS occurs in animal models of SAH [11]. The trigger for this upregulation is hypothesized to be increased shear stress on the endothelium as a result of the aneurysm, coupled with increased release of inflammatory mediators. At the same time, deficient immunoreactivity of NOS occurs following SAH in perivascular nerves, suggesting loss of nNOS after SAH [16]. This loss in nNOS is hypothesized to depend on oxidative stress following SAH, and subsequent development of BOX complexes derived from Hb [52].

Despite the loss of nNOS in vasospasm, it is known that nitrite/nitrate levels increase post-SAH [36], presumably due to increased expression of eNOS and iNOS following SAH [11, 53]. While this upregulation of NO production in the acute phase is critical for maintenance of cerebral blood flow in normal conditions, an uncoupling of NOS function from NO production may occur in pathological conditions, such as during SAH. Indeed, the presence of oxidative stress, the depletion of the cofactor tetrahydrobiopterin [14], and the presence of NOS inhibitors like ADMA [14, 54] have all been proposed as mechanisms that disrupt the flow of electrons within the NOS complex, leading to production of superoxide anion and peroxynitrite instead of NO [11]. This cascade of events represents targetable mechanisms that may exacerbate post-SAH oxidative stress and subsequently lead to vasospasm [10, 11, 53]. The biological effects of NOS, therefore, are quite complex. Much recent work has focused on whether or not modulation of this NOS dysfunction can prevent neurologic injury post-SAH. Proposed strategies will be addressed in the subsequent sections.

## 5. Treatment of Vasospasm

### 5.1. NO Donors in Vasospasm

Despite the controversies of NO dysfunction in CV and ischemic injury after SAH, several reports cite NO signaling as a potential therapy in preventing vasospasm. Sodium nitroprusside (SNP), a prodrug that is metabolized to NO, was the first approach utilized, with early animal studies confirming its vasodilatory effects. However, serious side effects of brain edema, hypotension, and the potential for cyanide toxicity led to discontinuation of this strategy as an intravenous approach [55]. Additional studies involving subarachnoid delivery of SNP in small numbers of patients demonstrated some vasodilatory potential, but the side effects of headache, nausea, vertigo, and rebound hypertension remained [56]. Intraventricular SNP has been attempted in some patients with medically refractory vasospasm. While these studies have small sample sizes, they demonstrate that SNP can improve indices of cerebral oxygenation such as brain oxygen tension and ultrasound-measured blood flow [57–61]. However, while intraventricular delivery of SNP improved evidence of vasospasm in some patients, it is not universally effective. Recent animal studies suggest that SNP fails to prevent vasospasm, despite biochemical evidence suggesting adequate delivery of the drug [62]. Therefore, current recommendations regarding SNP in vasospasm suggest that, given the potential consequences and uncertain efficacy, it should be considered for intraventricular administration only in patients that are otherwise medically refractory, mechanically ventilated, and deeply sedated [56]. 

IV nitroglycerin (GTN) is an additional NO donor approach, with rodent, canine, and primate studies demonstrating efficacy in preventing angiographic vasospasm [63–67]. The promising aspects of GTN therapy in vasospasm are confirmed in several smaller clinical studies [68–70]. However, the systemic side effects of this approach, such as systemic hypotension, drug tolerance, and rebound hypertension, prevent both SNP and GTN from becoming routine standard of care [71, 72].

Other studies involving GTN have looked at nonsystemic delivery approaches. Intrathecal approaches have been attempted in rabbit models, involving the implantation of pumps that deliver GTN continuously into the cerebromedullary cisterns, which prevent vasospasm while not affecting arterial blood pressure. However, this approach is not approved for use in humans and warrants further preclinical evaluation before clinical trials [73]. Intra-arterial delivery of GTN has also been described for treatment of vasospasm, though this was in patients who previously underwent superior temporal artery to internal carotid artery bypass graft. While intra-arterial GTN was effective in two patients who developed vasospasm in this study, it has not yet been determined to prevent vasospasm in the setting of SAH [72].

Given the systemic side effects of these approaches, new generations of NO donors have been developed, such as NONOates, S-nitrosothiols, and sodium nitrite. NONOates and S-nitrosothiols demonstrate efficacy in animal models but concern about systemic hypotension remain. Intrathecal/intracranial approaches have also demonstrated efficacy in animal models but concerns about potential adverse events preclude the development of human clinical trials [52, 74]. Based on recent observations that deoxyhemoglobin acts as a nitrite reductase enzyme [17], sodium nitrite is a particularly promising donor approach. Recent evidence in primate models suggests that IV sodium nitrite can prevent and reverse vasospasm and increase local CSF levels of NO [18]. The safety of sodium nitrite infusions was recently established in a clinical trial [19], suggesting that it can be infused intravenously at defined concentrations for prolonged intervals without a risk of systemic hypotension, an important finding compared to previous NO donors. Indeed, this approach has value for the study of ischemic damage in brain, as well as in the management of patients with organ transplants, blood-brain-barrier modulation, and pulmonary hypertension.

In addition to NO donors, recent evidence from animal models demonstrated the potential to target cGMP signaling itself for prevention of vasospasm. Intrathecal administration of a cell-membrane permeant form of cGMP prevented hemorrhage-associated hemodynamic changes in one rodent model [75]. Additionally, phosphodiesterase-5 inhibitors prevented vasospasm in rodent models [76, 77]. However, these approaches have yet to be explored in human studies.

### 5.2. Targeting NOS

The function of NOS is controlled by a combination of mechanisms including its transcription and phosphorylation. NOS activity is stimulated by phosphorylation from protein kinase C, calmodulin kinase II, Akt, AMPK, and others in response to upstream signals [78–80]. Given the role of both NO and NOS dysfunction in vasospasm, NOS emerges as an intriguing target to prevent vasospasm.

One such stimulator of NOS phosphorylation is the statin class of medications, drugs that target the protein HMG CoA-reductase. Multiple animal studies suggest that statins have benefits at preventing vasospasm [53, 81, 82]. One recent study indicated that statins prevent vasospasm through a mechanism involving recoupling of NOS function to NO production, and away from peroxynitrite production [53]. Randomized controlled trials also suggest that pravastatin improves outcomes after SAH [83]. Recent reviews on the topic of statins in CV agree that there is promising evidence to support statin therapy for prevention of delayed neurologic damage and improved outcomes; however, statins may not prevent vasospasm [84, 85]. Indeed, despite evidence from pooled randomized controlled trials, which suggest that delayed ischemic neurologic deficits are less in patients treated with statins, meta-analyses taking these RCTs into account with case control and observational studies remove this effect. Therefore, statins are not considered standard of care for treatment of CV patients [86].

Another interventional strategy to prevent vasospasm after SAH involves administration of erythtropoietin (EPO). EPO has a number of effects on blood vessels, and recent animal models suggest that adenoviruses expressing EPO protect against vasospasm through a mechanism depending on eNOS phosphorylation and modulation of survival signaling pathways like PI3K/Akt [87, 88]. Several different clinical trials have tried to translate these findings from animal models into practice. One such trial did not find an association between EPO and outcomes after SAH, although the authors of this trial suggest that a limited sample size may have contributed to these results. An additional trial had promising results including a reduction in the incidence of severe vasospasm, increased cerebral blood flow, and improved outcomes [88]. This trial additionally found a decreased need for transfusions and an increased reticulocyte count in patients receiving this treatment, consistent with the classic role of EPO in hematopoiesis [89]. The authors performed a follow up study to better understand the beneficial effects of EPO in CV, noting that EPO had a protective effect in younger patients and those without sepsis. Additionally, a trend toward improved outcomes in those also taking statins was seen [90]. A recent review suggested that EPO does not prevent vasospasm in clinical trials but may reduce the severity of vasospasm and improve outcomes [91]. EPO treatment in SAH still requires further investigation. Looking forward, recombinant forms of EPO that have a diminished erythropoietic effect but preserved neuroprotective effect hold promise for future therapies [92]. 

Many experimental approaches in animal models have aimed to modulate NOS in hopes of reducing vasospasm. Both moderate hypothermia and ischemic preconditioning, for example, are two approaches that alter the function of nitric oxide synthetic enzymes and have shown benefit in reducing vasospasm in animal models [39, 93]. Additionally, the hormone adiponectin, classically associated with fat cells, is also a potentially novel therapy to modulate NOS function. Adiponectin levels decrease after SAH and adiponectin administration stimulates eNOS phosphorylation [94].

Additional approaches to target NOS dysfunction itself involve genetic manipulation of NOS itself in both SAH and cardiovascular disease. Adenoviral mediated transfer of the eNOS gene has been attempted in preclinical models following injection of the viral inoculum into the CSF [95–97]. These experimental approaches suggest that the viral expression is limited to cerebral arteries and shows vasodilatory effects in SAH models [95–97]. Additional studies, involving *ex vivo *preparations of human cerebral blood vessels collected from patients undergoing temporal lobectomy, demonstrate that NOS gene transfer can also transform human cerebral blood vessels [98]. Indeed, these *ex vivo *preparations are also vasodilated by eNOS transfer, reflecting the effects of eNOS gene transfer in animal models [99]. However, while these findings are intriguing applications of gene therapy approaches, an eNOS gene transfer is difficult to develop a tightly controlled dose response-curve compared to a traditional pharmacological approach. This is particularly true with the adenoviral vectors used in the studies previously described, which will continue to express NOS protein long after the vasospastic phase. Indeed, while these considerations and others prevent clinical trials of NOS gene therapy, it is still an approach that has the potential for translation in the future, particularly if nonadenoviral vectors can be constructed that allow for more finely-tuned control of NOS gene expression. In conclusion, a summary of recent agents aimed at preventing CV using NO are seen in Table 1.

## 6. Conclusion

Two major hypotheses regarding the role of NO in SAH suggest that either a net defect in NO signaling, through the effects of Hb as an NO scavenger, or NOS uncoupling/free radical generation are mechanisms underlying vasospastic consequences. These theories are not mutually exclusive. Indeed, the function of ADMA, which is stimulated by Hb production and acts to uncouple NOS, provides a potential mechanism to rectify these hypotheses. Promising translational data for NO supplementation strategies stems from development of mechanisms for NO donation and infusions of sodium nitrite. Additionally, targeting NOS uncoupling and the neurodegenerative effects of reactive oxygen species, through administration of EPO, the statins, genetic manipulation of NOS, and other strategies, are intriguing and complementary strategies for treatment of SAH and other neurologic and vascular diseases. Future studies across clinical and animal models aimed at understanding non-CV mechanisms of neurologic deficits, and better assessment of NO itself, are warranted to aid in the understanding and treatment of vasospasm.

## Figures and Tables

**Table 1:** Experimental and clinical agents to prevent post-SAH vasospasm.

Agent	Mechanism	References	Summary
Sodium nitroprusside (SN)	NO donor	[56–62, 100–104]	(i) Potential to improve local hemodynamics in some animal model and human studies. (ii) Systemic hypotension, headache, nausea, vomiting, and potentially serious side effects of cyanide poisoning with both IV and intrathecal administration prevent routine use of SNP. (iii) Intraventricular administration of SNP may be warranted in medically refractory, mechanically ventilated, and deeply sedated patients.

Nitroglycerin (GTN)	NO donor	[63–66, 68–70]	(i) IV administration prevents CV in animal models including primate models. (ii) Transdermal approaches improve TCD values in clinical trials. (iii) Serious side effects of drug tolerance, systemic hypotension, and hypertensive rebound prevent routine use of systemic nitroglycerin in clinical setting. (iv) Intrathecal approaches effective in rabbit model, not approved in humans. (v) Intra-arterial approaches have been documented in 2 patients who suffered vasospasm of external carotid-internal carotid bypass grafts.

NONOates, S-nitrosothiols	NO donor	[52, 105]	(i) Cerebral vasodilator in SAH. (ii) Some adverse neurologic events in animal models. (iii) No clinical data available.

Sodium nitrite	NO donor	[17, 18, 20]	(i) Hemoglobin serves as nitrite reductase. (ii) Effective cerebral vasodilator in animal models. (iii) Prevents and reverses vasospasm in rodent and primate models without development of tolerance. (iv) Phase 1 trials demonstrate safety in humans.

Statins	Recoupling of NOS	[53, 81–86, 106]	(i) Efficacy at prevention of vasospasm in animal models. (ii) Discrepant clinical data regarding efficacy for vasospasm prevention. (iii) Clinical and animal data is promising, but not regarded as standard of care.

Erythropoietin (EPO)	Stimulates NOS signaling	[87–91]	(i) Efficacy at prevention of vasospasm in animal models. (ii) High doses needed for efficacy create clinical risk for thrombosis. (iii) Promising data from two clinical trials, but underpowered.

## Abbreviations

CV: Cerebral vasospasm
SAH: Subarachnoid hemorrhage.
